# Emergency Department Visit-Severity Algorithm for Immediate Care Clinic Visits

**DOI:** 10.5811/westjem.47360

**Published:** 2025-12-20

**Authors:** Jacy E. Neczypor, Talar W. Markossian, Luther Walls, Michael Cirone, Beatrice D. Probst

**Affiliations:** * Loyola University Chicago, Stritch School of Medicine, Maywood, Illinois; † Loyola University Chicago, Department of Public Health Sciences, Parkinson School of Health Sciences and Public Health, Maywood, Illinois; ‡ University of Illinois, Department of Family and Community Medicine, College of Medicine, Chicago, Illinois; § Advocate Christ Medical Center, Department of Emergency Medicine, Oak Lawn, Illinois; || Loyola University Medical Center, Department of Emergency Medicine, Maywood, Illinois

## Abstract

**Background:**

Immediate care clinics (ICC) account for a significant portion of acute, low-severity visits that preclude the use of resources from an emergency department (ED). Given the chronic issue of ED crowding and its detrimental effects on quality of care and health system efficiency, understanding and optimizing the use of ICCs for non-emergent visits could significantly alleviate pressures faced by EDs and improve patient satisfaction, as well as control the overall cost of care. This study describes the application of the Billings/Ballard severity algorithm to ICC visits over a seven-year period and compares the findings to previously published ED literature.

**Methods:**

We obtained data from ICC visits within a large, academic health system. The analytical sample included 306,395 visits from 125,063 unique patients. We describe ICC patient characteristics and the Billings/Ballard severity classification. We used negative binomial regression analysis to evaluate the associations between patient characteristics and total visits to ICCs and primary care physician (PCP), and multivariate regression analysis to assess the relationship between ICC visit severity and patient characteristics, controlling for multiple visits per patient. The algorithm was also used to identify and classify the most common International Classification of Diseases, 9th and 10th modifications (ICD-9/10) diagnosis codes by severity.

**Results:**

In total, 9.17% of ICC visits were classified as emergent, 81.25% as non-emergent, 0.79% as indeterminate, and 8.79% as unclassified, compared to literature-reported ED distributions of 37.90% emergent, 45.08% non-emergent, 11.32% indeterminate, and 5.70% unclassified. The ICC visits included a greater proportion of non-emergent presentations. The ICD-9/10 diagnosis distribution revealed a distinct ICC environment compared with that of the ED. The most frequent diagnoses among emergent ICC visits included chest pain, asthma exacerbation, and shortness of breath, while non-emergent visits were predominantly for upper respiratory tract infections. Within one year at the same healthcare system, 47% of patients had repeat ICC visits and 41% had primary care follow-up.

**Conclusion:**

These results demonstrate that immediate care clinics deliver predominantly non-emergent care as intended (81% vs 45% in the ED), potentially reducing ED crowding and validating current clinician- and patient-initiated referral practices. High rates of repeat ICC visits (47%) and follow-up with primary care physicians (41%) within the same healthcare system suggest these facilities foster care continuity while providing accessible, non-emergent care alternatives. However, user disparities persist as self-pay and uninsured patients show lower overall ICC use, while uninsured and publicly insured individuals present with emergent conditions more frequently than privately insured patients. These findings inform care-seeking education and health service delivery while highlighting the need to improve ICC accessibility across insurance types to optimize efficiency and patient outcomes.

## INTRODUCTION

Immediate care clinics (ICC), also known as urgent care centers, emerged in the United States in the 1970s and subsequently experienced rapid growth during the mid-2000s as part of a larger trend to deliver convenient, cost-effective medical services.^1^–^3^ The urgent care model has become a common source of timely and convenient care in the US healthcare system.^4^,^5^ To date, four models exist along the spectrum of consumer-driven care: immediate care clinics; free-standing emergency centers; retail or convenience clinics; and walk-in clinics.^2^ These alternate sources of care are not standardized in their delivery models but rather mutually aim to deliver patient care within the gap between physician office visits and hospital-based emergency departments (ED). Although the practice environments of emergency care, immediate care, and primary care are distinct, they sometimes overlap, and a myriad of factors influence patient decisions concerning when and where to choose care. For example, patients are often tasked with self-identifying time-sensitive conditions and balancing cost, convenience, insurance coverage, and location.^6^,^7^

In the US, causes of ED crowding include high costs of healthcare and access to other sources of care by uninsured and underinsured patients challenged in accessing a regular source of care that may be less costly.^8^–^11^ In theory, ICCs offer solutions to reduce specific system issues such as ED crowding, rising health expenditures, and access for patients who lack a usual source of care.^8^,^12^–^14^ However, while ICC charges for patients are often less expensive than those for an ED visit, there is evidence that immediate care use may increase net healthcare spending.^6^ Examining factors related to emergency care, immediate care, and primary care use has implications for shaping access to care (eg, meeting primary care needs and continuity of care), insurance provider operations (eg, guiding payees toward lower cost care), and healthcare businesses (eg, expanding healthcare systems operations).

Researchers at New York University developed an algorithm to characterize and assess the severity associated with ED visit diagnoses, which was subsequently validated by Ballard et al (2010) using data from a single, integrated delivery system that included members from a commercial insurance plan and Medicare.^15^,^16^ The Billings/Ballard algorithm has been revised and applied to describe the severity of ED visits in diverse settings.^17^–^19^ The paucity of ICC clinics in the late 1990s and early 2000s suggests that patients who now seek treatment in an ICC may have otherwise visited the ED during the development and validation of the Billings/Ballard algorithm. In addition, observation of ED crowding has led researchers to find that a proportion of patients could be treated more appropriately in an ICC setting.^8^,^20^–^22^ Taken together, these data suggest a likely overlap of cases between EDs and ICCs. Accurate triage based on case mix between ICC and ED environments is critical to optimizing resource use, maintaining timely access for high-acuity cases, and minimizing unnecessary healthcare expenditure.

**Population Health Research Capsule**

What do we already know about this issue?

*The Billings/Ballard algorithm is a previously validated emergency department (ED) severity algorithm that categorizes visits based on diagnosis codes*.

What was the research question?

*Can the Billings/Ballard algorithm characterize immediate care clinic (ICC) visits’ severity distribution patterns?*

What was the major finding of the study?

*Visits to ICCs were 81.25% non-emergent and 9.17% emergent vs ED rates of 45.08% and 37.90%, respectively*.

How does this improve population health?

*This evidence enables healthcare systems to optimize resource allocation and supports patient education for appropriate site selection, improving efficiency*.

Our objective in this study was to characterize ICC visits over a seven-year period, apply the Billings/Ballard severity algorithm to classify visit severity, and examine factors associated with ICC and primary care use. We analyzed patterns of ICC use including demographics, insurance status, and subsequent healthcare visits (ICC revisits, primary care physician [PCP] visits, and ED visits within 24 hours) to understand how ICCs function within the larger healthcare system. Additionally, we compared ICC severity distributions with those previously reported in the emergency medicine literature.

## METHODS

This was a retrospective review of data from five ICCs affiliated with a single, academic healthcare system between January 1, 2013–December 31, 2019. Our goal was to describe ICC visits over a seven-year period and describe the application of the Billings/Ballard algorithm to ICC visits. These ICCs are affiliated with a single, academic healthcare system that also includes two community hospitals, one university hospital, more than 20 primary care clinics, and three EDs located within a 50-mile radius. (Many other ICCs and EDs are also located within this 50-mile area.) We obtained the data used in this study from electronic health records. The Institutional Review Board for the Protection of Human Subjects deemed this study exempt. Our study adhered to key methodological standards for retrospective chart review research as outlined by Worster and Bledsoe including abstractor training, case selection criteria, variable definition, abstraction forms, blind to hypothesis, sampling method, missing-data management plan, and institutional review board approval.^23^

**Figure 1:**
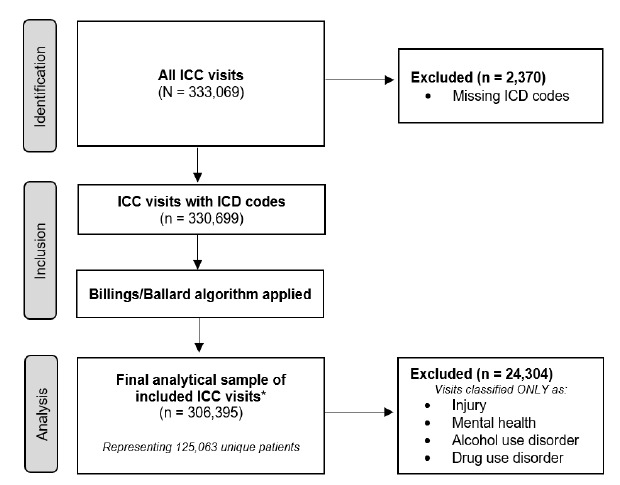
Flow diagram of immediate care clinic visit selection showing application of exclusion criteria for Billings/Ballard algorithm analysis. *Supplementary analysis comprises all visits with *ICD* codes (n = 330,699), including injury, mental health, alcohol use disorder, and substance use disorder categorizations. *ICC*, immediate care clinic; *ICD*, International Classification of Diseases.

The study population consisted of 333,069 visits to the ICCs. We excluded 2,370 (0.7%) visits for which *International Classification of Diseases*, 9^th^ and 10^th^ modifications (*ICD* 9/10) diagnosis codes were missing. Additionally, after the application of the Billings/Ballard algorithm and alignment with the original Billings/Ballard methodology, we excluded 24,304 (7.2%) visits that were associated with an injury, mental health, alcohol use disorder, or substance use disorder categorization only. Of these, most of the patients were excluded because of injury. The final analytical sample included 306,395 distinct visits to an ICC during the study period, corresponding to 125,063 unique patients (Figure 1). As injury could constitute a high diagnostic category for the ICC, we also calculated total visits to the ICC, including injury, mental health, and visits for alcohol use disorder and substance use order (Supplementary Table 1). In our study, the unit of analysis consisted of the ICC visit. In cases where visits were associated with multiple *ICD* 9/10 diagnosis codes, the *ICD code*s were aggregated at the visit level, and the severity of the ICC visit was associated with the most severe *ICD* code as classified by the Billings/Ballard algorithm.

### Variable and Measures

The primary outcome was ICC visit severity classification (emergent, non-emergent, or indeterminate) as determined by the Billings/Ballard algorithm. Secondary outcomes included healthcare use patterns following ICC visits such as ICC revisits, PCP visits, and ED referrals. Patient demographic characteristics (age, insurance status, race, ethnicity, sex) and prior healthcare use (total ICC and PCP visits) served as predictor variables in our analyses.

We queried patient demographic characteristics available from the EHR that have been shown to be associated with healthcare use. These included age, insurance status, race, ethnicity, and sex.^24^ We also queried the number of primary care visits, as this variable has previously been shown to be associated with urgent care use.^25^–^27^ Patient age was defined at the time of the ICC visit and categorized as < 18 years of age, 18–20.9, 21–25.9, 26–39.9, 40–64.9 years, or ≥ 65 years of age. Race (White, Black, Asian, other), ethnicity (non-Hispanic and Hispanic), and sex (female and male) were self-reported by the patients. Insurance status was classified as private, Medicare, Medicaid or other governmentally insured, self-paying or uninsured. We calculated revisits to any of the ICCs within 30 days, 90 days, six months and one year. The frequency of the same healthcare system PCP visits within 30 days, 90 days, six months, and one year after the ICC visit was also calculated. We also examined whether the ICC visit resulted in a same healthcare system ED referral within 24 hours. Finally, we calculated the total ICC and total PCP visits within the same healthcare system for a patient during the study period.

We classified ICC visits based on the original Billings algorithm methodology validated by Ballard et al (Supplementary Figure 1) using the discharge *ICD* 9/10 diagnosis codes. According to this algorithm, each *ICD* 9/10 code is assigned a likelihood probability for each of the following categories: 1) non-emergent; 2) emergent, primary care treatable; 3) emergent, ED care needed and preventable; and 4) emergent, ED care needed and not preventable.^16^ We compared the sum of the probabilities of the non-emergent and emergent primary care-treatable categories to the sum of the emergent ED care needed (preventable) and emergent ED care needed (not preventable) categories.^15^ The largest likelihood probability determined the final classification of a visit as non-emergent or emergent. Visits with an equal probability of being non-emergent or emergent were categorized as indeterminate. According to this classification scheme, we classified ICC visits into emergent, non-emergent, and indeterminate categories. Like Ballard et al, we excluded visits classified as injury-only or “other.” Given that a single visit may contain multiple *ICD* diagnosis codes, ICC visits are categorized by the most severe *ICD* 9/10 code according to the algorithm. The classification of visits into one of three categories (non-emergent, indeterminate, emergent) by Ballard et al has been demonstrated to reliably predict hospitalization and death within 30 days following an ED visit.^15^

### Statistical Analysis

We used univariate analysis to analyze the characteristics of the ICC visits and subsequent ICC, PCP, and ED visits. We described both the severity of the ICC visits over the study period and the payor type. We applied negative binomial regression analysis to examine the association between patient characteristics and the total number of ICC and PCP visits over the study period. The results are presented as incident rate ratios (IRR) with confidence intervals. Negative binomial regression appropriately accounts for over-dispersed count outcome variables, such as the number of ICC and PCP visits during the study period. The unit of analysis for these regressions was the unique patients who visited the ICC during the study period. These analyses controlled for the number of times each patient visited the ICC. We then performed random effects logistic regression analysis for odds ratios (OR) and 95% confidence intervals to examine factors associated with emergent visits compared to non-emergent visits while controlling for multiple visits per patient. Missing data were included in the regression analysis as a separate category (missing data were < 1–3% for all variables); however, these data are not reported in Table 1. We performed all analyses using Stata 17 (StataCorp LLC, College Station, TX). The statistical significance was assessed at the α = 0.05 level.

## RESULTS

Table 1 displays population characteristics and same healthcare system use for patients at multiple, suburban ICCs in Illinois from 2013–2019. No significant changes in patient descriptive characteristics were found across the study timeframe; as a result, we present data for all study years combined. The median number of ICC visits by patient was 4 (interquartile range 2, 8) over seven years. There were 9.19%, 20.8%, 33.18%, and 47.56% of patients who revisited an ICC within a 30-day, 90-day, six-month, and one-year period, respectively; and 7.89%, 19.93%, 30.88%, and 41.14% of patients who visited their PCP within a 30-day, 90-day, six-month, and one-year period, respectively. Additionally, 1.9% of patients presented to an ED of the same healthcare system within 24 hours of their initial ICC encounter.

Table 2 shows the results of the Billings/Ballard algorithm applied to the ICC setting. The distributions within each of the severity categories remained constant over the study period and are reported as seven-year totals. Of 306,395 ICC visits, 91.21% were classified by the algorithm. We found that 9.17% of the patients were classified as emergent, 0.79% as indeterminate, and 81.25% as non-emergent.

Table 3 classifies the severity categories by payor type to examine differences in ICC use by insurance coverage. The association between the Billings/Ballard algorithm and payor type was statistically significant (*P* < .001). Within each payor category, 15.67% of Medicare visits were classified as emergent, followed by 9.31% of uninsured or self-paying visits, 8.38% of privately insured visits, and 7.84% of Medicaid and other governmental insurance visits. Conversely, within each payor category, 82.68% of Medicaid and other governmental insurance visits were classified as non-emergent, followed by 82.14% of privately insured visits, 75.52% of uninsured or self-paying visits, and 74.62% of Medicare visits.

Table 4 displays the factors associated with the total number of ICC and PCP visits in the same healthcare system during the study period. For the model with total ICC visits as the outcome variable, identifying as male (IRR 0.98; CI, 0.98–0.99), being > 40 years of age (IRR 0.99; CI, 0.98–0.99) or > 65 years of age (IRR 0.94; CI, 0.92–0.96), and identifying as White (IRR 0.94; CI, 0.94–0.95), Asian (IRR 0.91; CI, 0.88–0.93), or other (IRR 0.93; CI, 0.92–0.95) were associated with a decreased incidence of ICC use. Identification as Hispanic was associated with a greater incidence of ICC use (IRR 1.05; CI, 1.04–1.06). Compared to patients with private insurance, patients with Medicare (IRR 1.04; CI 1.02–1.07) or Medicaid (IRR 1.11; CI, 1.10–1.12) were associated with a greater incidence of ICC presentation, while uninsured and self-pay patients were associated with a decreased incidence of ICC presentation (IRR 0.93; CI, 0.90–0.95). A positive association between patient PCP visits and ICC presentation was observed within the same healthcare system.

With respect to the model in which total PCP visits were the outcome variable, all age groups, compared to those 26–39.9 years of age, demonstrated a positive association with PCP visits, where the highest incidence rate of PCP presentation was for patients < 18 years of age (IRR 3.06; CI, 2.94–3.18) and > 65 years of age (IRR 3.00; CI, 2.78–3.23). Being White (IRR 1.37; CI, 1.32–1.43), Asian (IRR 1.40; CI, 1.27–1.54), and identifying as “other” (IRR 1.39; CI, 1.31–1.47) race was associated with more frequent PCP visits than Black identification. Identification as Hispanic was associated with decreased incidence of PCP visits (IRR 0.65; CI, 0.63–0.68). Compared to patients with private insurance, patients insured by Medicare were more likely to have a greater incidence of PCP presentation (IRR 1.64; CI, 1.53–1.75), while those who were uninsured or self-pay were more likely to have a decreased incidence of PCP presentation (IRR 0.43; CI, 0.39–0.48). There was no difference in the incidence of PCP visits between patients who were covered by Medicaid and those with private insurance coverage.

Table 5 shows factors associated with ICC presentation for emergent visits compared to non-emergent visits. Being male (OR 1.27; CI, 1.23–1.31), 40–64.9 years of age (OR 1.44; CI, 1.37–1.51), ≥ 65 years (OR 1.73, CI 1.59, 1.89), identifying as Hispanic (OR 1.12; CI,1.06–1.18), having Medicare (OR 1.44; CI, 1.33–1.56) or Medicaid (OR 1.09; CI, 1.0–1.14) coverage, and being self-paying or uninsured (OR 1.14; CI 1.00–1.30) were significantly associated with increased odds of ICC presentation for an emergent visit. Factors significantly associated with decreased odds of emergent visits to the ICC included younger age, < 18 years (OR 0.61; CI, 0.58–0.65), 18–20.9 years of age (OR 0.71; CI, 0.64–0.78), or 21–25.9 years of age (OR 0.83; CI, 0.76–0.90), identifying as White (OR 0.75; CI, 0.71–0.79) or Asian (OR 0.67; CI, 0.59–0.75), and presenting to the ICC more than once, 2–3 ICC visits (OR 0.90; CI, 0.8–0.94), 4–5 ICC visits (OR 0.86; CI, 0.82–0.91), and 6–9 ICC visits (OR 0.93; CI, 0.88–0.98). The same healthcare system PCP visit, irrespective of the frequency of PCP visits, was positively associated with the frequency of an emergent visit in patients within the same healthcare system.

Supplementary Table 1 displays a comparison of the results of the Billings/Ballard algorithm applied in an ICC setting with those of an ED inclusive of injuries, alcohol use disorder, substance use disorder, and mental health-related visits, where injuries are reported separately. The larger denominator in this supplementary analysis (N = 330,699 vs 306,395 in the main analysis) reflects the inclusion of visits classified as injury, mental health, alcohol use disorder, or substance use disorder that were excluded from the primary analysis. According to our data, 15.27% of the ICC visits were associated with an injury *ICD* 9/10 code. In Supplementary Table 2, we present the five most common *ICD* codes according to the Billings/Ballard severity categorizations. The most frequent emergent *ICD* 9/10 diagnosis codes were for chest pain, asthma exacerbation, shortness of breath, pneumonia, and acute obstructive laryngitis. The most frequent non-emergent *ICD* 9/10 codes included acute pharyngitis, upper respiratory tract infections, cough, streptococcal pharyngitis, and acute bronchitis.

## DISCUSSION

These results characterize ICC use patterns within a suburban academic healthcare system and demonstrate distinct severity distributions between ICCs and EDs, while also revealing the dynamics between ICCs, EDs, and PCPs. Further iterations of the Billings/Ballard algorithm have been applied within the ED environment to obtain a more systematic perspective of visit characteristics, including the addition of a separate injury category with subcategorizations, as well as mental health, alcohol use disorder-, and substance use disorder-related visits.^19^ Our initial analysis focused on the original Billings/Ballard algorithm to demonstrate a proof of concept in applying the algorithm in the ICC setting, but we also provide a comparison of a derivative of the Billings/Ballard algorithm (Supplementary Table 1). While Ballard et al reported 37.90% emergent ED visits in their integrated delivery system (Table 2), applying the Billings/Ballard algorithm to nationally representative National Hospital Ambulatory Medical Care Survey data yielded 13.43% emergent visits as reported by Lemke et al (2020).^15^,^19^ Our ICC emergent rate of 9.17% is lower than reported ED rates in the literature, suggesting that ICCs function as intended to serve lower acuity conditions.

Non-emergent visits constituted a greater proportion of visits in the ICC than in the ED. Among ICC visits, 9.17% were classified as emergent and 81.25% as non-emergent, in contrast to 37.90% and 45.08%, respectively, among ED visits reported in the literature.^15^ Notably, ICCs accounted for a much smaller proportion of indeterminate visits (0.79%) than EDs (11.32%), possibly reflecting patients’ self-triage decisions for ambiguous or uncertain symptoms. The ICCs were primarily used for lower acuity conditions, particularly respiratory infections, and were less likely to receive patients with conditions more common in EDs, such as wounds, syncope, abdominal pain, and urinary tract infections (Supplementary Table 2).^15^ Additionally, visit severity varied by patient demographics and payor characteristics, whereby uninsured or self-pay patients were underrepresented in both ICC and primary care visits.

To our knowledge, this is the first study to apply and describe the Billings/Ballard algorithm in the setting of immediate care clinics, and it addresses a notable gap in the literature. The successful classification of 91.21% of visits across a large, demographically diverse patient population demonstrates the algorithm’s applicability to ICC environments. While this evidence shows that ICCs predominantly serve privately insured patients (70.28% vs 66.5% nationally), the finding that uninsured patients were less likely to use either ICCs or PCPs highlights persistent access disparities in non-emergency, acute care settings.^28^,^29^,^33^ Additionally, the positive association between PCP use and ICC visits within our healthcare system challenges the concern that ICCs disrupt primary care relationships, suggesting instead that these settings may function as complementary components of integrated care delivery.^25^,^26^ These patterns, combined with the distinct severity distributions between ICCs and EDs, provide essential data for optimizing acute-care resource allocation.

The clinical implications of these findings are particularly relevant to emergency medicine. Clearer delineation between the case mix of ICCs and EDs may facilitate more appropriate patient self-triage and reduce the burden of low-acuity visits on strained EDs as well as improve the allocation of emergency care resources. Furthermore, the observed associations between visit severity, patient characteristics, and insurance status offer actionable insights for clinicians and healthcare system leaders aiming to align acute care delivery with clinical urgency. It also highlights opportunities to reduce healthcare costs by supporting site-appropriate care and aligning with evolving reimbursement structures.

This study establishes the feasibility of applying the Billings/Ballard algorithm to ICC settings, providing a standardized method for comparing severity distributions across acute care environments. Our findings highlight the importance of distinguishing the roles of ICCs and EDs to promote appropriate patient use, reduce avoidable ED burden, and support the delivery of timely, high-acuity emergency care. These pre-pandemic data demonstrate the algorithm’s utility in classifying ICC visits and provide a reproducible framework for characterizing ICC acuity patterns in other healthcare systems and time frames.

## LIMITATIONS

Despite the strengths of including data from multiple ICCs and primary care clinics associated with an academic healthcare system, a large sample of diverse patient populations, and multiple years of data, this study has limitations. The examination of the severity of ICC visits at five suburban ICCs in Illinois associated with an academic medical center may not be representative of healthcare systems in other states or across the country. Moreover, the ICCs spanned into suburban regions of a large metropolitan area, which may or may not be applicable to other areas across the country. Demographic and geographic differences may exist for other ICCs not affiliated with an academic medical center. Furthermore, *ICD* 9/10 coding errors and the novel application of the Billings/Ballard algorithm to ICC settings may have led to misclassification of visit severity, potentially biasing our regression estimates. However, the high classification rate (91.21%) and clinically meaningful patterns observed support the algorithm’s utility despite these potential limitations.

## CONCLUSION

This study demonstrates the successful application of the Billings/Ballard algorithm to immediate care clinic settings. Compared to previously published ED severity category distributions, we found a lower proportion of emergent and indeterminate visits and a greater proportion of non-emergent visits within the ICC, revealing that ICCs predominantly serve their intended population of patients with lower acuity conditions. A review of the most frequent diagnosis codes in the ICC and ED revealed distinct conditions comprising the emergent and non-emergent categories in each setting. In addition, we found disparate patterns of ICC use based on various patient characteristics, including age, race, ethnicity, and insurance status. Our results help to characterize the same healthcare system ICC usage at an academic medical center, as well as the interplay between PC and ED settings.

By delineating the role of ICCs within the acute care landscape, our study provides evidence to guide healthcare system optimization in access, efficiency, and cost control. For emergency physicians, these findings support confident referral of stable, low-acuity patients to ICCs, potentially alleviating ED crowding and allowing focus on higher acuity cases. Healthcare system administrators can use these data to optimize resource allocation and develop evidence-based triage protocols. For policymakers, the disparities in ICC use by insurance status highlight the need for targeted interventions to improve access for uninsured and underinsured populations. Public health initiatives should communicate these severity differences through patient-facing tools that guide appropriate selection of care site, ultimately improving healthcare efficiency, patient satisfaction, and cost effectiveness.

## Supplementary Information







## Figures and Tables

**Table 1:** Patient characteristics and healthcare use at multiple suburban immediate care clinics in a study applying an emergency department severity algorithm to immediate care clinic visits (N= 306,395; 2013–2019).

Patient characteristics	2013–2019	

	Frequency (n)	Percentage (%)^a^
Number of ICC Visits per patient: median; IQR; range	2; IQR 1–4; range 1–71	
Number of PCP Visits per patient: median; IQR; range	0; IQR 0–9; range 0–232	
Sex		
Female	183,726	59.96%
Male	122,666	40.04%
Age		
< 18 years	99,823	32.58%
18 – 20.9 years	11,487	3.75%
21 – 25.9 years	17,668	5.77%
26 – 39.9 years	47,933	15.64%
40 – 64.9 years	92,104	30.06%
≥ 65 years	37,024	12.08%
Race		
White	215,962	70.57%
Black	41,323	13.50%
Asian	5,529	1.81%
Other^b^	35,313	11.54%
Ethnicity		
Non-Hispanic	245,006	80.02%
Hispanic	54,693	17.86%
Insurance Status		
Private	215,349	70.28%
Medicare	36,400	11.88%
Medicaid and other governmentally insured	50,810	16.58%
Self-pay and uninsured	3,836	1.25%
Revisits to ICC (cumulative)		
Within 30 days	28,185	9.19%
Within 90 days	63,749	20.80%
Within 6 months	101,688	33.18%
Within 1 year	145,744	47.56%
Visit to PCP after ICC visit (cumulative)		
Within 30 days	24,185	7.89%
Within 90 days	61,088	19.93%
Within 6 months	94,628	30.88%
Within 1 year	126,080	41.14%
ED Visit		
Within 24 hours	5,848	1.90%

a Column percentages may not total 100% due to missing values in some of the variables.

b Other race categories include Alaska Native, Native American, multiracial, Native Hawaiian, and other Pacific Islander.

*ED*, emergency department; *ICC*, immediate care clinic; *IQR*, interquartile range; *PCP*, primary care physician.

**Table 2:** Comparison of the Billings/Ballard algorithm applied to immediate care clinic visits and emergency department (ED) visits in a study applying an ED severity algorithm to immediate care clinic visits.

Ballard Algorithm Visit Classification	Data from current ICC study (N = 306,395)	Ballard et al (ED)^a^,^b^
Emergent	9.17%	37.90%
Indeterminate	0.79%	11.32%
Non-emergent	81.25%	45.08%
Unclassified	8.79%	5.70%
Total	100.00%	100.00%

a Data source: Medical Care. 2010;48(1):58–63. https://doi.org/10.1097/MLR.0b013e3181bd49ad.^15^

b Original Ballard percentages were based on total classified visits. Percentages were recalculated to include unclassified visits.

*ED*, emergency department; *ICC*, immediate care clinic.

**Table 3:** Billings/Ballard algorithm classification by type of payor in a study applying an emergency department severity algorithm to immediate care clinic visits^a^ (N = 306,395; 2013–2019).

Percentage distribution by type of payor				

Ballard algorithm classification	Medicare	Medicaid	Private	Uninsured/Self-Pay
Emergent	15.67	7.84	8.38	9.31
Indeterminate	1.19	0.92	0.68	0.99
Non-emergent	74.62	82.68	82.14	75.52
Unclassified	8.52	8.56	8.79	14.18
Total (%)	100	100	100	100

a Chi-squared test significant, *P* < .001.

**Table 4:** Multivariable regression analysis examining the association between patient demographics, primary care physician visits, and total immediate care clinic use in a study applying an emergency department severity algorithm to immediate care clinic visits^a^ (N = 125,063; 2013–2019).

	Total PCP^b^ visits Incident rate ratio (IRR) (95% CI)	Total ICC visits Incident rate ratio (IRR) (95% CI)
Sex (Ref. Female)		
Male	0.87 *** (0.85, 0.90)	0.98 *** (0.98, 0.99)
Age at first ICC visit (Ref. 26 – 39.9 years)		
< 18 years	3.06 *** (2.94, 3.18)	1.01 (0.99, 1.02)
18 – 20.9 years	2.28 *** (2.12, 2.45)	1.01 (0.99, 1.03)
21 – 25.9 years	1.60 *** (1.51, 1.70)	1.00 (0.99, 1.02)
40 – 64.9 years	2.13 *** (2.05, 2.21)	0.99 * (0.98, 0.99)
≥ 65 years	3.00 *** (2.78, 3.23)	0.94 *** (0.92, 0.96)
Race (Ref. Black)		
White	1.37 *** (1.32, 1.43)	0.94 *** (0.94, 0.95)
Asian	1.40 *** (1.27, 1.54)	0.91 *** (0.88, 0.93)
Other^c^	1.39 *** (1.31, 1.47)	0.93 *** (0.92, 0.95)
Ethnicity (Ref. Non-Hispanic)		
Hispanic	0.65 *** (0.63, 0.68)	1.05 *** (1.04, 1.06)
Insurance status at first ICC visit (Ref. Private)		
Medicare	1.64 *** (1.53, 1.75)	1.04 *** (1.02, 1.07)
Medicaid and other governmentally insured	1.04 (1.00, 1.08)	1.11 *** (1.10, 1.12)
Self-pay and uninsured	0.43 *** (0.39, 0.48)	0.93 *** (0.90, 0.95)
Total PCP visits at the same health system (Ref. 0)		
1 – 4	-----------------------------	1.07 *** (1.06, 1.08)
5 – 12	-----------------------------	1.08 *** (1.06, 1.09)
13 – 29	-----------------------------	1.10 *** (1.09, 1.11)
30 – 232	-----------------------------	1.13 *** (1.11, 1.14)

* *P* < .05,

** *P* < .01,

*** *P* < .001.

a Negative binomial regression, regressions controlled for number of years patient has visited the ICC.

b Same healthcare system primary care physician.

c Other race categories include Alaska Native, Native American, multiracial, Native Hawaiian, and other Pacific Islander.

*ICC*, immediate care clinic; *IRR*, incident rate ratio; *IQR*, interquartile range; *PCP*, primary care physician.

**Table 5:** Multivariate regression analysis examining patient demographics and healthcare use associated with emergent vs non-emergent immediate care clinic (ICC) visits in a study applying an emergency department severity algorithm to ICC visits^a^ (N= 306,395; 2013–2019).

	Odds ratio (95% CI)
Sex (Ref. Female)	
Male	1.27 *** (1.23, 1.31)
Age (Ref. 26–39.9 years)	
<18 years	0.61 *** (0.58, 0.65)
18 – 20.9 years	0.71 *** (0.64, 0.78)
21 – 25.9 years	0.83 *** (0.76, 0.90)
40 – 64.9 years	1.44 *** (1.37, 1.51)
≥ 65 years	1.73 *** (1.59, 1.89)
Race (Ref. Black)	
Asian	0.67 *** (0.59, 0.75)
Other^a^	0.79 *** (0.73, 0.85)
White	0.75 *** (0.71, 0.79)
Ethnicity (Ref. Non-Hispanic origin)	
Hispanic origin	1.12 *** (1.06, 1.18)
Insurance type (Ref. Private)	
Medicare	1.44 *** (1.33, 1.56)
Medicaid, other governmentally insured	1.09 *** (1.03, 1.14)
Self-Pay and uninsured	1.14 * (1.00, 1.30)
Total ICC visits (Ref. 1 visit, 17.4%)	
2–3 visits (26.23%)	0.90 *** (0.86, 0.94)
4–5 visits (18.3%)	0.86 *** (0.82, 0.91)
6–9 visits (20.35%)	0.93 ** (0.88, 0.98)
10–71 visits (17.66%)	1.00 (0.94, 1.06)
Total PCP visits at the same health system (Ref. 0)	
1–4	1.10 *** (1.05, 1.15)
5–12	1.16 *** (1.11, 1.22)
13–29	1.21 *** (1.15, 1.27)
30–232	1.36 ** (1.28, 1.44)

* *P* < .05,

** *P* < .01,

*** *P* < .001.

a Random-effects logistic regression analysis; the regression analysis controlled for multiple visits per patient.

b Other race categories include Alaska Native, Native American, multiracial, Native Hawaiian, and other Pacific Islander.

*ICC*, immediate care clinic; *PCP*, primary care physician.
