# Supplementary material for: Emergency Department Visit-Severity Algorithm for Immediate Care Clinic Visits
Source: West J Emerg Med. 2025 Dec 20;27(1):184–93. doi: 10.5811/westjem.47360 (PMC12815509; doi:10.5811/westjem.47360)
Supplement: Supplementary file 1 [file wjem-27-184-s001.docx]

| Supplementary Table 1. Comparison of the Billings/Ballard algorithm between immediate care clinic and emergency department settings, where injuries are reported separately, in a study applying an emergency department severity algorithm to immediate care clinic visits | | |
| --- | --- | --- |
| **Ballard Algorithm Visit Classification** | **Data from current ICC study (n=330,699)** | **ED**^a^ |
| Emergent | 7.97% | 13.43% |
| Indeterminate | 0.69% | 1.21% |
| Non-emergent | 67.75% | 47.74% |
| Injury^b^ | 15.27% | 20.30% |
| Psych, Alcohol, Drug Use | 0.16% | 3.62% |
| Unclassified | 8.14% | 13.69% |
| Total | 100.00% | 100.00% |
| ^a^Data Source: Am J Manag Care. 2020;26(3):119-125. https://doi.org/10.37765/ajmc.2020.42636^19^  ^b^Injury classification comprises any visit associated with an injury ICD code. | | |
