# Supplementary material for: Emergency Department Visit-Severity Algorithm for Immediate Care Clinic Visits
Source: West J Emerg Med. 2025 Dec 20;27(1):184–93. doi: 10.5811/westjem.47360 (PMC12815509; doi:10.5811/westjem.47360)
Supplement: Supplementary file 2 [file wjem-27-184-s002.docx]

| Supplementary Table 2. Rank by frequency, the top five ICD codes per Billings/Ballard severity categorizations in a study applying an emergency department severity algorithm to immediate care clinic visits^a^ (N= 585,579; 2013-2019) | |
| --- | --- |
| **Emergent (10.05% of all classified ICC visits)** | |
| **ICD9/10 Code(s)**^b^ | **Diagnosis Description** |
| R079/78650 | Chest pain, unspecified |
| JJ45901/J4521/49392 | Asthma, unspecified type, with acute exacerbation |
| R0602 | Shortness of breath |
| J189 | Pneumonia, unspecified organism |
| J050 | Acute obstructive laryngitis (croup) |
| **Non-emergent (89.08% of all classified ICC visits)** | |
| J029/462 | Acute pharyngitis, unspecified |
| J069 | Acute upper respiratory infection, unspecified |
| R05 | Cough |
| J020 | Streptococcal pharyngitis |
| J209 | Acute bronchitis, unspecified |
| **Indeterminate (0.86% of all classified ICC visits)** | |
| J219 | Acute bronchiolitis, unspecified |
| H1131/H1132 | Conjunctival hemorrhage, left/right eye |
| 49121 | Obstructive chronic bronchitis with (acute) exacerbation |
| 46619 | Acute bronchiolitis due to other infectious organisms |
| H538 | Other visual disturbances |
| ^a^One visit may be associated with more than one ICD.  ^b^Categories based on ICD-9 and ICD-10 diagnoses. | |
