# Supplementary material for: Emergency Department Visit-Severity Algorithm for Immediate Care Clinic Visits
Source: West J Emerg Med. 2025 Dec 20;27(1):184–93. doi: 10.5811/westjem.47360 (PMC12815509; doi:10.5811/westjem.47360)
Supplement: Supplementary file 3 [file wjem-27-184-s003.docx]

| Supplementary Figure 1. Classification of immediate care clinic visits according to the 2000 Billings NYU algorithm in a study applying an emergency department severity algorithm to immediate care clinic visits^15^ (n=306,395) |
| --- |
|  |
| *Exclusive of ICC visits that had only injury, mental health, alcohol use, or substance use related diagnoses.  NE=Non-emergent PCT= Primary Care Treatable EPA= Emergency care needed, preventable/avoidable  ENPA= Emergency care needed, nonpreventable/avoidable |
